# *Capparis spinosa* Attenuates Hepatic Inflammation and Fibrosis by Suppressing Inflammatory Cytokines in a Wistar Rat Model of Nonalcoholic Steatohepatitis

**DOI:** 10.5812/ijpr-166099

**Published:** 2025-11-04

**Authors:** Rasoul Akbari, Tahereh Behdarvand, Hamid Yaghooti, Mohammad Taha Jalali, Narges Mohammadtaghvaei

**Affiliations:** 1 Hyperlipidemia Research Center, Ahvaz Jundishapur University of Medical Sciences, Ahvaz, Iran; 2 Department of Laboratory Sciences, School of Allied Medical Sciences, Ahvaz Jundishapur University of Medical Sciences, Ahvaz, Iran; 3 Aligoudarz School of Nursing, Lorestan University of Medical Science, Khorramabad, Iran; 4 Department of Clinical Biochemistry, Faculty of Medical Sciences, Tarbiat Modares University (TMU), Tehran, Iran

**Keywords:** Nonalcoholic Steatohepatitis, *Capparis spinosa*, TNF-α, IL-6, Adiponectin, Leptin

## Abstract

**Background:**

Non-alcoholic fatty liver disease (NAFLD) is a prevalent chronic hepatic condition that can progress to non-alcoholic steatohepatitis (NASH) through inflammatory processes.

**Objectives:**

This research aimed to examine the impact of an aqueous extract of *Capparis spinosa* fruit and the lipid-lowering agent fenofibrate (FENO) on hepatic inflammation and steatosis in rats subjected to a high-fat emulsion.

**Methods:**

Male Wistar rats were given a high-fat diet (HFD) to develop NASH. The high-fat treated rats were categorized into three groups and administered either *C. spinosa*, FENO, or a vehicle control. Histopathological analyses, Liver Index computation, and measurements of body and liver weights were conducted. Serum levels of liver enzymes, adiponectin, and leptin were also assessed. Additionally, the expression of hepatic genes for monocyte chemoattractant protein 1 (MCP-1), transforming growth factor-beta (TGF-β), interleukin 6 (IL-6), and tumor necrosis factor-alpha (TNF-α) was evaluated.

**Results:**

The administration of *C. spinosa* extract to the NASH model rodents significantly increased their adiponectin levels while substantially decreasing their levels of leptin, alanine aminotransferase (ALT), and aspartate transaminase (AST). Hepatic steatosis, liver inflammation, and collagen deposition were significantly reduced by *C. spinosa* treatment. Furthermore, the hepatic mRNA expression of the proinflammatory cytokines TNF-α, IL-6, and MCP-1, as well as the hepatic fibrogenic marker TGF-β1, was significantly reduced by *C. spinosa* treatment. The FENO exhibited a comparable pattern of response.

**Conclusions:**

Our findings suggest that *C. spinosa* has a positive anti-inflammatory effect and may protect the liver against hepatic fibrosis, inflammation, and steatosis. These findings demonstrate the promising therapeutic potential of *C. spinosa* in the management of NASH.

## 1. Background

In recent years, non-alcoholic fatty liver disease (NAFLD) has become one of the most common chronic liver diseases in developed countries (1). This condition is often linked to metabolic issues like type 2 diabetes and obesity. A major feature of NAFLD is low-grade inflammation, which is closely related to insulin resistance and the buildup of fat in the liver. A more severe form of this disease, called non-alcoholic steatohepatitis (NASH), is marked by significant inflammation in the liver (2). If NASH is not treated, it can lead to serious problems such as cirrhosis and liver fibrosis, mainly due to the release of pro-inflammatory substances from both fat tissue and immune cells in the liver (3).

Cytokines like tumor necrosis factor-alpha (TNF-α), interleukin 6 (IL-6), monocyte chemoattractant protein 1 (MCP-1), and transforming growth factor-beta (TGF-β) are important players in the development of NASH (4). When liver macrophages become activated, they release these inflammatory and fibrotic factors such as TGF-β, which then stimulate hepatic stellate cells. These stellate cells are crucial in the process of fibrosis, creating a connection between inflammation and fibrosis in the liver (5, 6). The MCP-1 is produced by various types of liver cells, including hepatocytes and activated stellate cells, highlighting its key role in liver inflammation and its progression to fibrosis (7). On the other hand, IL-6 has a more complicated role in liver disease, especially in disrupting insulin signaling (8).

In addition to these cytokines, important signaling molecules called adipokines, such as adiponectin and leptin, also play significant roles in NAFLD and NASH. Adiponectin helps improve insulin sensitivity and protects the liver by reducing inflammation and fibrosis. It has been shown to help reduce liver fat and inflammation by lowering levels of TNF-α in the liver and in the bloodstream (9-11). While leptin is mainly produced by fat tissue, it can also be made by activated stellate cells during inflammation (12). In the early stages of NAFLD, leptin may help reduce fat buildup in the liver, but in later stages of NASH, it tends to promote inflammation and fibrosis (13).

Despite the development of various treatments in recent years, there is still no widely accepted cure for NAFLD/NASH. This has led to increased interest in finding effective medications for these conditions. Herbal remedies that have antioxidant, cholesterol-lowering, and blood sugar-lowering properties may help relieve symptoms of NAFLD. Traditional medicine around the world often uses herbal extracts to treat liver diseases (14). One such plant is *Capparis spinosa*, which belongs to the *Capparidaceae* family and has been shown to have cholesterol-lowering, antioxidant, and anti-inflammatory effects. Research has found that *C. spinosa* extract can significantly lower blood sugar and cholesterol levels in diabetic rats. Additionally, *C. spinosa* is known to contain anti-inflammatory compounds that can reduce the production of pro-inflammatory substances (1, 15, 16).

## 2. Objectives

This study aims to explore the protective effects of *C. spinosa* fruit extract on liver tissue and inflammation markers related to NASH in mice fed a high-fat diet (HFD). Given the reported benefits of this plant, the research will also compare the effects of the extract with those of fenofibrate (FENO), a medication known for its positive effects on cholesterol levels and fatty liver. The study will focus on how *C. spinosa* affects the expression of liver genes related to inflammation and fibrosis.

## 3. Methods

### 3.1. Materials

The FENO and a vitamin-mineral formulation were procured from Abidi Pharmaceutical Company, Tehran, Iran. The high-fat emulsion comprised sodium deoxycholate, corn oil, total milk powder, cholesterol, Tween 80, and propylene glycol, with all components supplied by Sigma-Aldrich Corporation, United States.

### 3.2. Preparation of the Capparis spinosa Aqueous Extract

*Capparis spinosa* specimens were collected in Shoosh (Khuzestan province, Iran) in June 2020. A botanical specialist recognized the specimens and deposited them in the herbarium of the Ahvaz Jundishapur University of Medical Sciences in Ahwaz, Iran. The aqueous extract of *C. spinosa* was produced according to the techniques described by Jalali et al. (1). *Capparis spinosa* fruits were cleansed with distilled water and dried at 40°C before being crushed into a powder. The aqueous extract was made by immersing 10 g of powdered fruits in 100 milliliters of distilled water and carefully mixing for 2 hours. The mixture was subsequently allowed to settle for 20 minutes after boiling for 15 minutes. In order to eliminate particulate matter, the aqueous extract was filtered through a 0.2 mm Millipore filter (Millipore, St Quentin en Yvelines, France). The filtrate that was obtained was freeze-dried and stored at -20°C for future use. The aqueous extracts were produced daily, just before dosing. Jalali et al. (1) reported that the extract was prepared by reconstituting freeze-dried extracts in 1.5 mL of distilled water and administered orally to the experimental groups by gavage for six weeks at a dosage of 20 mg/kg body weight once a day.

### 3.3. Preparation of High-Fat Emulsion

In accordance with the Zou et al. formulation, the high-fat emulsion diet contained 77% calories from fat, 9% from carbohydrates, and 14% from proteins (2). This therapy administered saccharose, maize oil fat, and complete milk powder proteins as carbohydrates. The high-fat emulsion's makeup is as follows: 400 g corn oil; 150 g saccharose; 80 g complete milk powder; 100 g cholesterol; 10 g sodium deoxycholate; 1.5 g mineral combination; 36.4 g Tween 80; 2.5 g vitamin mix; 31.1 g propylene glycol; 10 g culinary salt; and 300 mL purified water. Originally kept at 4°C, this emulsion was painstakingly blended in a 42°C water immersion prior to use. The animals received the high-fat emulsion diet via gavage at 10 mL/kg/day.

### 3.4. Animals, Treatments, and Experimental Design

The Experimental Animal Center at Ahvaz Jundishapur University of Medical Sciences provided 36 mature male Wistar rats, with an average weight of 198 ± 11 g. Rats were housed in plastic enclosures with uniform environmental conditions. These comprised a 12-hour light/12-hour dark cycle, a constant temperature of 25 ± 2℃, and humidity levels of 55 - 60%. They were given a typical rat chow diet and unrestricted access to drinking water prior to the commencement of the study. All operations were authorized by the Ahvaz Jundishapur University of Medical Sciences Research Center and Experimental Animal House Ethics Committee. Following adaptation, rats were randomly assigned to either a normal control (NC) group (n = 9) or a HFD group (n = 27) using a random number table. The NC group received the normal diet. The high-fat emulsion (10 mL/kg) was administered to the HFD group by gavage at 9:00 a.m. for 12 weeks while maintaining the same diet. Equivalent volumes of saline solution were given to the NC group. Furthermore, the HFD group was given unrestricted access to a saccharose solution (18%). The HFD group rats received streptozotocin intraperitoneally during the fifth week after it had been dissolved in 0.2 mL of 0.1 M citrate buffer, pH 4.5. The NASH model phenotype, which is defined by obesity and immobility, was observed in the HFD group during the sixth week. One rat from the control group and one rat from the HFD group were sacrificed to verify the development of NASH. A pathologist evaluated their livers in accordance with the histological criteria for NASH. Using a random number system, the NASH model group was split up into three groups at the start of the seventh week: (1) The HFD control group that received the high-fat emulsion, (2) the group that received 10 mg/kg body weight HFD + *C. spinosa*, and (3) the group that received the HFD+FENO 100 mg/kg body weight. *Capparis spinosa *and FENO were suspended in 0.5% carboxymethylcellulose-Na (CMC) solution and given to the animals via gavage daily at 5:00 PM until the end of the 12th week.

### 3.5. Collection of Liver Tissues and Blood Samples

After a 12-hour fast at the conclusion of the 12-week study, all animals were anesthetized using a combination of xylazine (10 mg/kg) and ketamine hydrochloride (90 mg/kg) prior to euthanasia. Blood samples were collected via cardiac puncture, and serum was separated for the analysis of serum leptin, adiponectin, and biochemical markers. The rat livers were promptly excised and rinsed with standard cold saline. Specimens were weighed to determine the Liver Index, then frozen in liquid nitrogen (-180°C) for further investigation of hepatic gene expression. In all groups, a liver specimen was preserved in 10% formalin for histological examination.

### 3.6. Histopathological Examination

Collagen deposition was evaluated using Masson's trichrome, while hepatic lipid accumulation (steatosis) was investigated using hematoxylin and eosin (H&E). These histological alterations were evaluated in 7 mm liver sections using standard staining methods. The slices were then examined under a light microscope. Images were captured using a Leica ICC50 HD digital camera attached to a motorized light microscope. The degree of inflammation, fibrosis stage, and steatosis region were assessed using predetermined criteria.

### 3.7. Biochemical Parameters Analysis

#### 3.7.1. Liver Function Tests

Blood levels of alanine transaminase (ALT) and aspartate aminotransferase (AST) were measured using the Roche 6000 auto-analyzer with appropriate test kits (Roche, catalog number: 4718569190 and 465754390, respectively).

#### 3.7.2. Serum Leptin and Adiponectin

Serum levels of adiponectin and leptin were determined using a commercial ELISA kit from R&D Systems (Minneapolis, MN, USA).

#### 3.7.3. RNA Extraction, Real-time Polymerase Chain Reaction, and Hepatic Gene Expression

The hepatic gene expressions of TGF-β1, TNF-α, IL-6, and MCP-1 were quantified using quantitative polymerase chain reaction (qPCR). Total RNA was extracted from the frozen liver samples using TRIzol^™^ Reagent (Invitrogen, Grand Island, NY) according to the manufacturer's instructions. The integrity and concentration of the total RNA were assessed using spectrophotometry (Nanodrop ND-1000; Thermo Scientific). The cDNA was synthesized using Avian Myeloblastosis Virus (AMV) Reverse Transcriptase (Takara Bio, Otsu, Japan).

Quantitative real-time PCR (qRT-PCR) analysis was conducted using the SYBR Green PCR reagent (Takara Bio, Otsu, Japan) on the Gentier96E 3 Real-Time PCR System (Tianlong Science and Technology), following the manufacturer's instructions. The comparative threshold cycle method was used to analyze hepatic gene expression levels, which were normalized using β-actin as an endogenous control gene (relative quantification using ΔΔCt methods). Gene expression data were analyzed using Applied Biosystems software. Primer pairs sequences used for the RT-PCR reactions are shown in Table 1. 

**Table 1.** Sequence of the Primers Used for Real-time PCR

Primer Name	Forward Primer	Reverse Primer
**TGF-β1**	5′-CAAAGACATCACACACAGTA-3′	5′-GGTGTTGAGCCCTTTCCAGG-3′
**TNF-α**	5′-ACCACGCTCTTCTGTCTACTG-3′	5′-CTTGGTGGTTTGCTACGAC-3′
**MCP-1**	5'-GTGCTGACCCCAATAAGGAA -3′	5'-TGAGGTGGTTGTGGAAAAGA-3′
**IL-6**	5'- TGATGGATGCTTCCAAACTG-3′	5'- GAGCATTGGAAGTTG GGGTA-3′
**β-actin**	5′-CCCATCTATGAGGGTTACGC-3′	5′-TTTAATGTCACGCACGATTTC-3′

Abbreviations: TGF-β, transforming growth factor-beta; TNF-α, tumor necrosis factor-alpha; MCP-1, monocyte chemoattractant protein 1; IL-6, interleukin 6.

### 3.8. Statistical Analysis

The data were presented as the mean ± standard deviation (SD). One-way ANOVA was used, followed by Tukey’s post-hoc test for multiple comparisons, using GraphPad Prism version 8.0.2 for Windows (GraphPad Software, La Jolla, CA, USA). The significance threshold for all statistical tests was set at P < 0.05.

## 4. Results

### 4.1. Effects of Capparis spinosa on Body Weight and Liver Index in Rats Fed with the High-fat Diet

**Figure 1.**
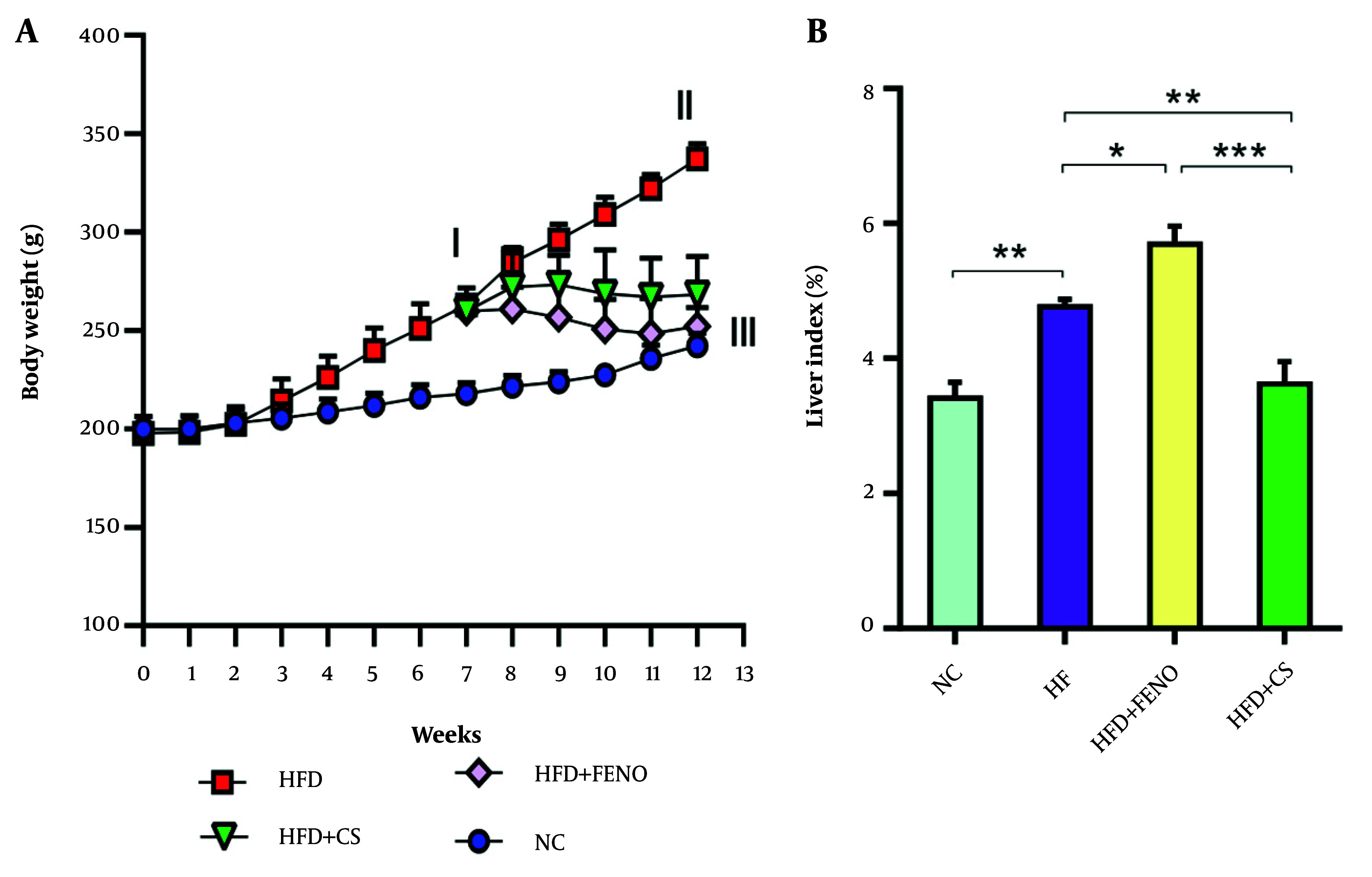
Effect of *Capparis spinosa* fruit extract and fenofibrate (FENO) on body weight (A) and percentage of Liver Index (B). Bars represent the mean ± standard deviation (SD) of the variables in each group [n = 7 - 8; CS: *Capparis spinosa*; Abbreviations: NC, normal control; HFD, high-fat diet; * P < 0.05, ** P < 0.01, and *** P < 0.001; (I) Significantly different from NC at the end of week 6, (II) significantly different from NC after 12 weeks, and (III) significantly different between NC, HFD+FENO, and HFD+*C. spinosa* vs. HFD at the end of week 12 (P < 0.001)].

Rats administered the HFD for 12 weeks exhibited significantly higher final Body Weights and Liver indices (liver weight/body weight × 100%) compared to those in the NC group (P < 0.01, Figure 1A and B). The increase in body weight was normalized after six weeks of *C. spinosa* with FENO medication (P < 0.001). Furthermore, the Liver Index demonstrated a substantial decline in response to the *C. spinosa* after six weeks of therapy (P < 0.01, Figure 1B). In contrast, the Liver Index was not only not diminished by FENO medication but was also significantly increased compared to the HFD group (P < 0.05).

### 4.2. Effect of Capparis spinosa on Serum Alanine Aminotransferase, Aspartate Transaminase, Leptin, and Adiponectin Levels in Rats Fed with the High-fat Diet

**Figure 2.**
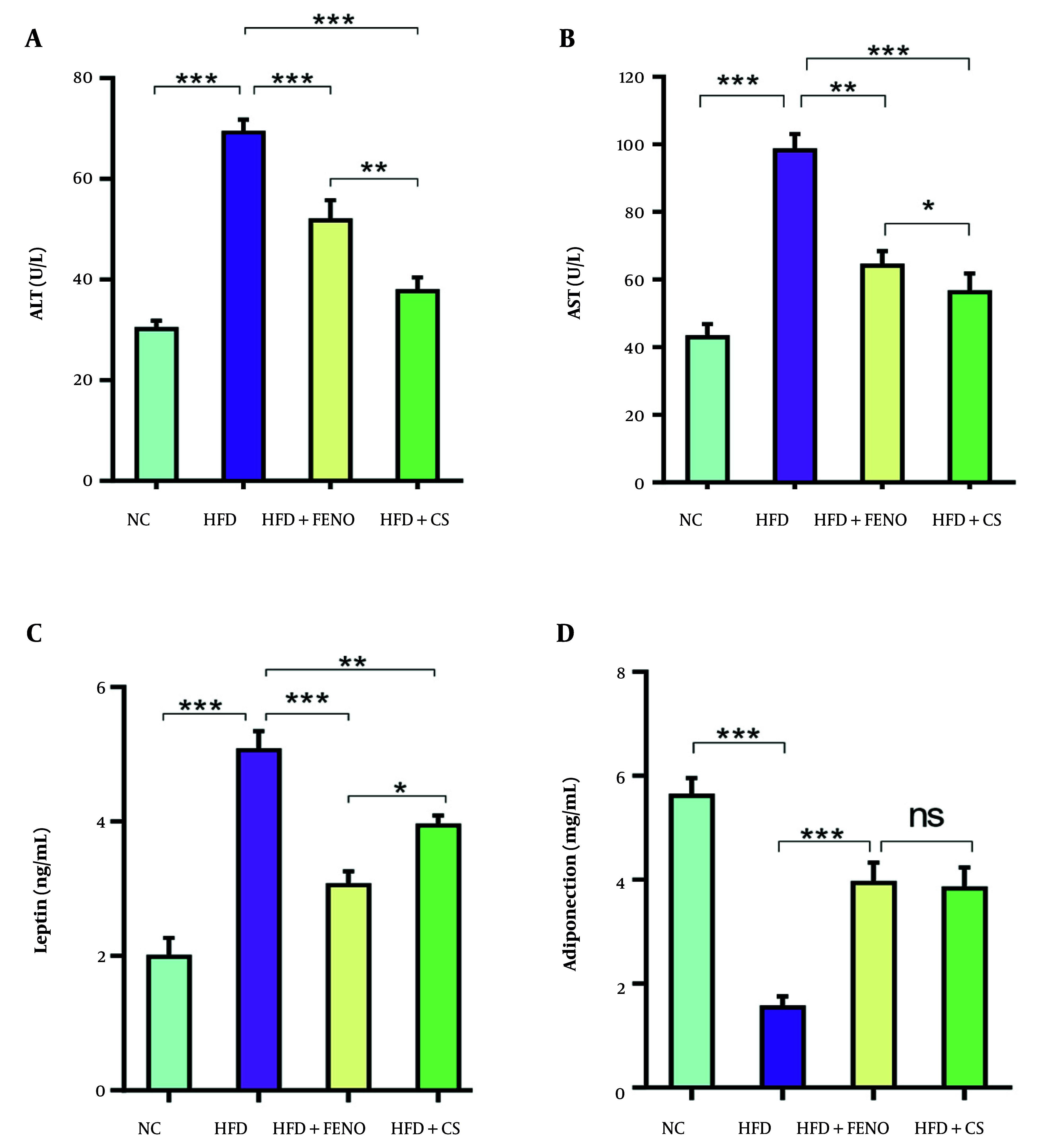
Effects of *Capparis spinosa* extract on serum alanine aminotransferase (ALT), aspartate transaminase (AST), leptin, and adiponectin: (A) serum ALT, (B) serum AST, (C) serum leptin, and (D) serum adiponectin in rats fed a high-fat diet (HFD). Values are expressed as the mean ± standard deviation (SD, n = 7 rats; between-group comparisons were tested by ANOVA followed by Tukey Kramer multiple comparisons test; CS: *Capparis spinosa*; abbreviations: NC, normal control; FENO, fenofibrate; ns, non-significant; * P < 0.05, ** P < 0.01, and *** P < 0.001).

Figure 2 shows that the HFD group had significantly higher serum levels of ALT and AST than the NC group (P < 0.001). Compared to the HFD group, the *C. spinosa*-treated group's blood levels of AST and ALT were considerably lower (P < 0.001, Figure 2A and B). Serum AST and ALT levels were also decreased by FENO therapy; however, the *C. spinosa* effect was much greater (P < 0.001, Figure 2A and B). Insulin resistance and related disorders, such as obesity and NAFLD, have been linked to adiponectin and leptin. Therefore, we assessed the degree to which *C. spinosa* affected blood levels of adiponectin and leptin. The HFD-fed animals had significantly greater blood leptin levels than the NC group (P < 0.001, Figure 2C). However, *C. spinosa* treatment partially corrected the rise in blood leptin levels caused by the HFD (P < 0.01, Figure 2C). The HFD mice's blood adiponectin levels were significantly lower than the NC group's (P < 0.001, Figure 2D). However, *C. spinosa* and FENO treatments could dramatically raise the adiponectin levels that were markedly lowered after the HFD was administered.

### 4.3. Effects of Capparis spinosa on Hepatic Steatosis, Liver Inflammation, and mRNA Expression of Proinflammatory Cytokines in the Liver of High-fat Diet-Fed Rats

**Figure 3.**
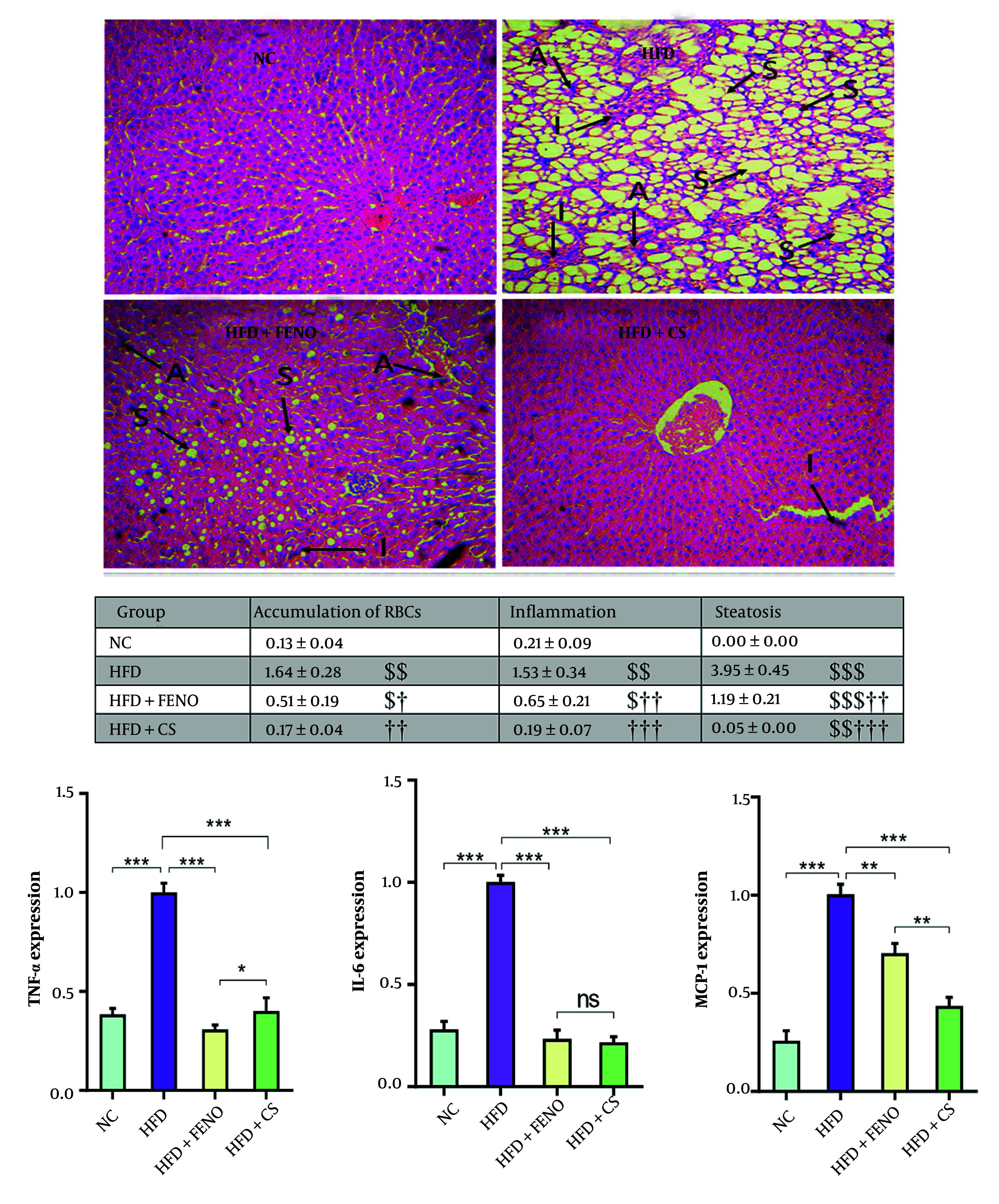
*Capparis spinosa* extract reduces high-fat diet (HFD)-induced hepatic steatosis and inflammation in the non-alcoholic steatohepatitis (NASH) model (n = 7 - 8). Upper panel: Histopathological observations of liver sections stained with hematoxylin and eosin (H&E, magnification: 100X; abbreviations: A, accumulation of RBCs; I, inflammation; S, steatosis); middle panel: Mean ±  standard deviation (SD) of quantitative histopathological assessment for steatosis, accumulation of RBCs, and inflammation after H&E staining; lower panel: The expression levels of inflammation-related genes in rats fed a HFD (CS: *Capparis spinosa*; abbreviations: TNF-α, tumor necrosis factor-alpha; IL-6, interleukin 6; MCP-1, monocyte chemoattractant protein 1; NC, normal control; FENO, fenofibrate; ns, non-significant; ** P < 0.01 and *** P < 0.001; $ P < 0.05, $$ P < 0.01, and $$$ P < 0.001 vs. NC; † P < 0.05, †† P < 0.01, and ††† P < 0.001 vs. HFD).

The H&E staining was used to investigate the impact of *C. spinosa* extract on hepatic lipid accumulation and inflammation. Unlike the NC group, which exhibited significant steatosis in liver cells, the HFD-fed group showed advanced degrees of inflammation, marked by enlarged hepatocytes, extensive cytoplasmic fat vacuoles, and small lipid droplets (Figure 3, upper panel). Additionally, the HFD diet led to a significant accumulation of erythrocytes in the liver tissue (Figure 3, middle panel). However, *C. spinosa* treatment resulted in a substantial reduction in hepatic inflammation and lipid accumulation compared to the HFD-fed group. Furthermore, the micro- and macrovesicular steatosis and hepatic inflammation induced by HFD were diminished in response to FENO treatment (Figure 3, middle panel). Consequently, there was a substantial increase in the mRNA expression of proinflammatory cytokines IL-6, TNF-α, and MCP-1 in HFD-fed rats compared to healthy rats (P < 0.001, Figure 3, lower panel). Conversely, the mRNA levels of these cytokines were significantly reduced by *C. spinosa* treatment compared to the HFD group (P < 0.001). Animals administered FENO exhibited comparable outcomes.

### 4.4. Effects of Capparis spinosa on Hepatic Fibrosis and mRNA Expression of Transforming Growth Factor-Beta 1 in the Liver of High-fat Diet-Fed Rats

**Figure 4.**
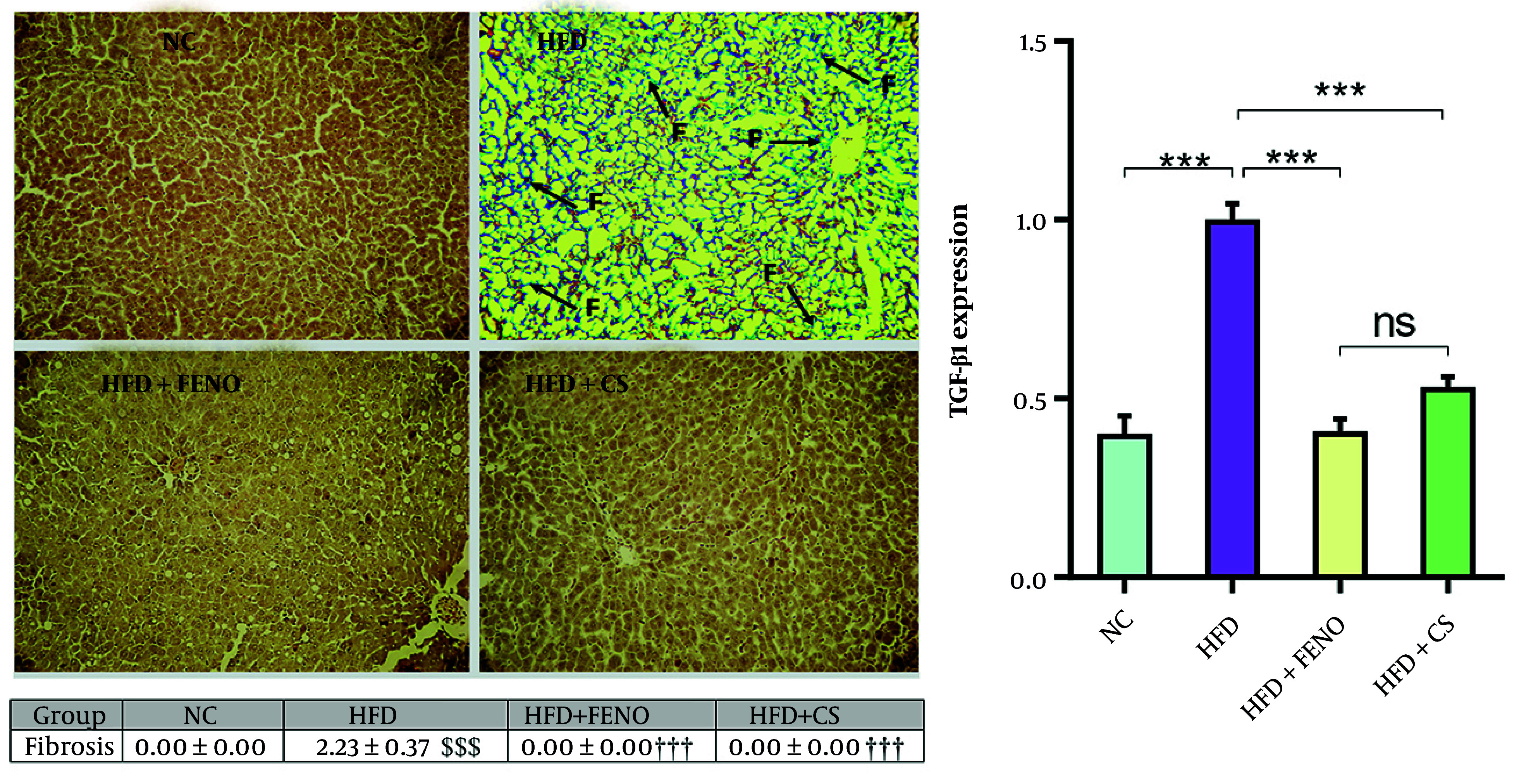
*Capparis spinosa* extract improves high-fat diet (HFD)-induced hepatic fibrosis in the non-alcoholic steatohepatitis (NASH) model. Upper panel: Representative images of Masson Trichrome stained liver tissue sections (magnification: 100X; abbreviation: F, fibrosis); middle panel: Quantitative histopathological assessment for fibrosis and collagen deposition; lower panel: Expression of hepatic transforming growth factor-beta (TGF-β) 1 mRNA in rats fed a HFD. Values are given as mean  ±  standard deviation (SD, CS: *Capparis spinosa*; Abbreviations: NC, normal control; FENO, fenofibrate; n = 7 - 8 rats; *** P < 0.001; $$$ P < 0.001 vs. NC; ††† P < 0.001 vs. HFD).

The liver sections of rodents in the HFD group exhibited a significant amount of collagen deposition and fibrosis, as illustrated in Figure 4. Masson's trichrome staining was used to confirm this. The combination of *C. spinosa* and FENO for 42 days substantially decreased hepatic fibrosis and collagen deposition (P < 0.001, Figure 4, upper and middle panels). The HFD group had significantly higher levels of TGF-β1 mRNA expression than the NC group, corroborating this finding. The TGF-β1 mRNA expression decreased considerably with *C. spinosa* and FENO treatment (P < 0.001, lower panel).

## 5. Discussion

The results of this study provide strong evidence for the therapeutic potential of *C. spinosa* in treating NASH induced by a HFD. Our findings demonstrate that *C. spinosa* treatment significantly reduced liver enzyme levels, specifically ALT and AST, which are critical indicators of hepatocellular injury. This protective effect was more pronounced than that observed with FENO, suggesting that *C. spinosa* may offer superior hepatoprotection. The reduction in these enzymes indicates that *C. spinosa* can effectively shield hepatocytes from damage, likely due to its antioxidant properties, which have been previously documented in various studies (15-17).

In addition to lowering liver enzyme levels, *C. spinosa* treatment resulted in a notable decrease in body weight and Liver Index compared to the HFD group. This is particularly relevant considering the established link between obesity, insulin resistance, and the progression of NAFLD to NASH. The weight reduction observed in the *C. spinosa*-treated group aligns with findings from earlier studies that highlight the herb’s role in modulating lipid metabolism and promoting weight loss. The mechanism behind this effect may involve the regulation of genes associated with lipogenesis and lipolysis, which *C. spinosa* has been shown to influence, thereby improving metabolic health (18). The FENO-treated group also exhibited weight loss benefits, despite the fact that the Liver Index increased rather than decreased. Animal studies have demonstrated that hepatomegaly is one of the adverse effects of FENO in rodents, which is consistent with our findings (19).

The study also revealed that *C. spinosa* treatment significantly decreased levels of pro-inflammatory cytokines, including TNF-α, IL-6, and MCP-1. Elevated levels of these cytokines are well-known contributors to the inflammatory processes that exacerbate liver damage in NASH. For instance, TNF-α has been implicated in promoting insulin resistance and activating inflammatory pathways, including nuclear factor-B and c-jun N-terminal kinase, that lead to hepatocyte injury (20). Studies suggest a correlation between hepatic fibrosis and TNF-α levels in patients with NASH (21). Our results indicate that *C. spinosa* may inhibit the production of TNF-α, thereby reducing the inflammatory response and protecting the liver. This is consistent with previous research that has demonstrated the anti-inflammatory effects of *C. spinosa* in various models of metabolic disorders (22, 23).

Moreover, the reduction in MCP-1 levels observed in the *C. spinosa*-treated rats is particularly significant, as MCP-1 is a key chemokine that facilitates the recruitment of inflammatory cells to the liver, contributing to the progression of hepatic inflammation and fibrosis (24, 25). Our findings support earlier studies that have shown a correlation between elevated MCP-1 levels and the severity of liver disease. The ability of *C. spinosa* to normalize MCP-1 levels suggests a mechanism by which it mitigates inflammation and fibrosis in the liver.

The modulation of adipokines by *C. spinosa* is another critical aspect of our findings. The observed decrease in serum leptin levels, coupled with an increase in adiponectin, highlights the herb’s role in restoring adipokine balance in the context of metabolic syndrome. Leptin is known to promote inflammation and insulin resistance (26), while adiponectin exerts protective effects on the liver by enhancing insulin sensitivity and exhibiting anti-inflammatory properties (27). Leptin, through the glucose-stimulated insulin secretion pathway, is directly related to the secretion of insulin granules, which may promote insulin resistance (28). It has been shown that leptin increases TGF-β in Kupffer cells, which causes fibrogenesis and inflammation in the liver (12). The *C. spinosa* treatment resulted in a decrease in leptin levels in these animals. This result was also consistent with the reduction in body weight. The changes in these adipokines following *C. spinosa* treatment align with previous findings that link low adiponectin levels to the development of NAFLD/NASH.

Furthermore, the significant reduction in TGF-β1 expression following *C. spinosa* treatment underscores its anti-fibrotic potential. The TGF-β1 is a well-established mediator of fibrogenesis, and its upregulation is associated with increased collagen production and liver fibrosis (29, 30). Our results indicate that *C. spinosa* can effectively downregulate TGF-β1, thereby potentially reversing the fibrotic process. This finding is consistent with studies that have highlighted the importance of targeting TGF-β1 in the management of liver fibrosis.

Histological analysis further corroborated our biochemical findings, showing a marked improvement in liver architecture in the *C. spinosa*-treated group. The reduction in steatosis, inflammatory cell infiltration, and collagen deposition observed in liver sections aligns with the biochemical improvements noted. Previous studies have shown that effective treatments for NASH often lead to similar histological outcomes (31, 32), reinforcing the notion that *C. spinosa* may serve as a viable therapeutic option for managing liver disease.

### 5.1. Conclusions

In conclusion, our study presents compelling evidence that *C. spinosa* aqueous extract offers significant protective effects against HFD-induced NASH. The herb’s ability to modulate key inflammatory cytokines, improve adipokine profiles, and reduce liver injury markers suggests that it could be a promising alternative treatment for NASH. Given the multifactorial nature of the disease, further investigation into the precise mechanisms of action and potential clinical applications of *C. spinosa* is warranted, particularly in the context of developing effective therapies for metabolic liver diseases.

### 5.2. Limitations

This study has not determined which of the active compounds of the *C. spinosa* is responsible for these effects, and it is necessary to examine this in future studies.

## Data Availability

The datasets used and/or analyzed during the current study are available from the corresponding author upon reasonable request.
